# Practical applications of soil microbiota to improve ecosystem restoration: current knowledge and future directions

**DOI:** 10.1111/brv.13124

**Published:** 2024-07-29

**Authors:** Shawn D. Peddle, Riley J. Hodgson, Ryan J. Borrett, Stella Brachmann, Tarryn C. Davies, Todd E. Erickson, Craig Liddicoat, Miriam Muñoz‐Rojas, Jake M. Robinson, Carl D. Watson, Siegfried L. Krauss, Martin F. Breed

**Affiliations:** ^1^ College of Science and Engineering Flinders University Sturt Road Bedford Park South Australia 5042 Australia; ^2^ SoilsWest, Centre for Sustainable Farming Systems, Food Futures Institute Murdoch University 90 South Street Murdoch Western Australia 6150 Australia; ^3^ University of Waikato Te Whare Wananga o Waikato Gate 1 Knighton Road Hamilton 3240 New Zealand; ^4^ Department of Biodiversity, Conservation and Attractions Kings Park Science Kattidj Close Kings Park Western Australia 6005 Australia; ^5^ Centre for Engineering Innovation, School of Agriculture and Environment The University of Western Australia Stirling Highway Crawley Western Australia 6009 Australia; ^6^ Department of Plant Biology and Ecology University of Seville C. San Fernando Sevilla Spain; ^7^ School of Biological, Earth and Environmental Sciences, Centre for Ecosystem Science University of New South Wales Sydney New South Wales 2052 Australia; ^8^ School of Biological Sciences The University of Western Australia Stirling Highway Crawley Western Australia 6009 Australia

**Keywords:** ecosystem restoration, improved ecological outcomes, positive soil legacy, recovery trajectory, restoration genomics, restoration methods

## Abstract

Soil microbiota are important components of healthy ecosystems. Greater consideration of soil microbiota in the restoration of biodiverse, functional, and resilient ecosystems is required to address the twin global crises of biodiversity decline and climate change. In this review, we discuss available and emerging practical applications of soil microbiota into (*i*) restoration planning, (*ii*) direct interventions for shaping soil biodiversity, and (*iii*) strategies for monitoring and predicting restoration trajectories. We show how better planning of restoration activities to account for soil microbiota can help improve progress towards restoration targets. We show how planning to embed soil microbiota experiments into restoration projects will permit a more rigorous assessment of the effectiveness of different restoration methods, especially when complemented by statistical modelling approaches that capitalise on existing data sets to improve causal understandings and prioritise research strategies where appropriate. In addition to recovering belowground microbiota, restoration strategies that include soil microbiota can improve the resilience of whole ecosystems. Fundamentally, restoration planning should identify appropriate reference target ecosystem attributes and – from the perspective of soil microbiota – comprehensibly consider potential physical, chemical and biological influences on recovery. We identify that inoculating ecologically appropriate soil microbiota into degraded environments can support a range of restoration interventions (e.g. targeted, broad‐spectrum and cultured inoculations) with promising results. Such inoculations however are currently underutilised and knowledge gaps persist surrounding successful establishment in light of community dynamics, including priority effects and community coalescence. We show how the ecological trajectories of restoration sites can be assessed by characterising microbial diversity, composition, and functions in the soil. Ultimately, we highlight practical ways to apply the soil microbiota toolbox across the planning, intervention, and monitoring stages of ecosystem restoration and address persistent open questions at each stage. With continued collaborations between researchers and practitioners to address knowledge gaps, these approaches can improve current restoration practices and ecological outcomes.

## I. INTRODUCTION

Overexploitation of natural systems has led to the biodiversity crisis (Ceballos *et al*., 2015; Dirzo & Raven, 2003) and vast areas of degraded ecosystems (Gibbs & Salmon, 2015). While conserving remnant ecosystems is a priority, there is also a need to restore degraded areas to biodiverse and functioning ecosystems (Higgs *et al*., 2018; Perring, Erickson & Brancalion, 2018; Moreno‐Mateos *et al*., 2020). Accordingly, there is a marked increase in ecosystem restoration globally, with targets to restore more than 350 million hectares under The Bonn Challenge and the United Nations declaring 2021–2030 the Decade on Ecosystem Restoration. However, there is considerable room to improve the success of restoration projects (Crouzeilles *et al*., 2016; Wortley, Hero & Howes, 2013).

**Fig. 1:**
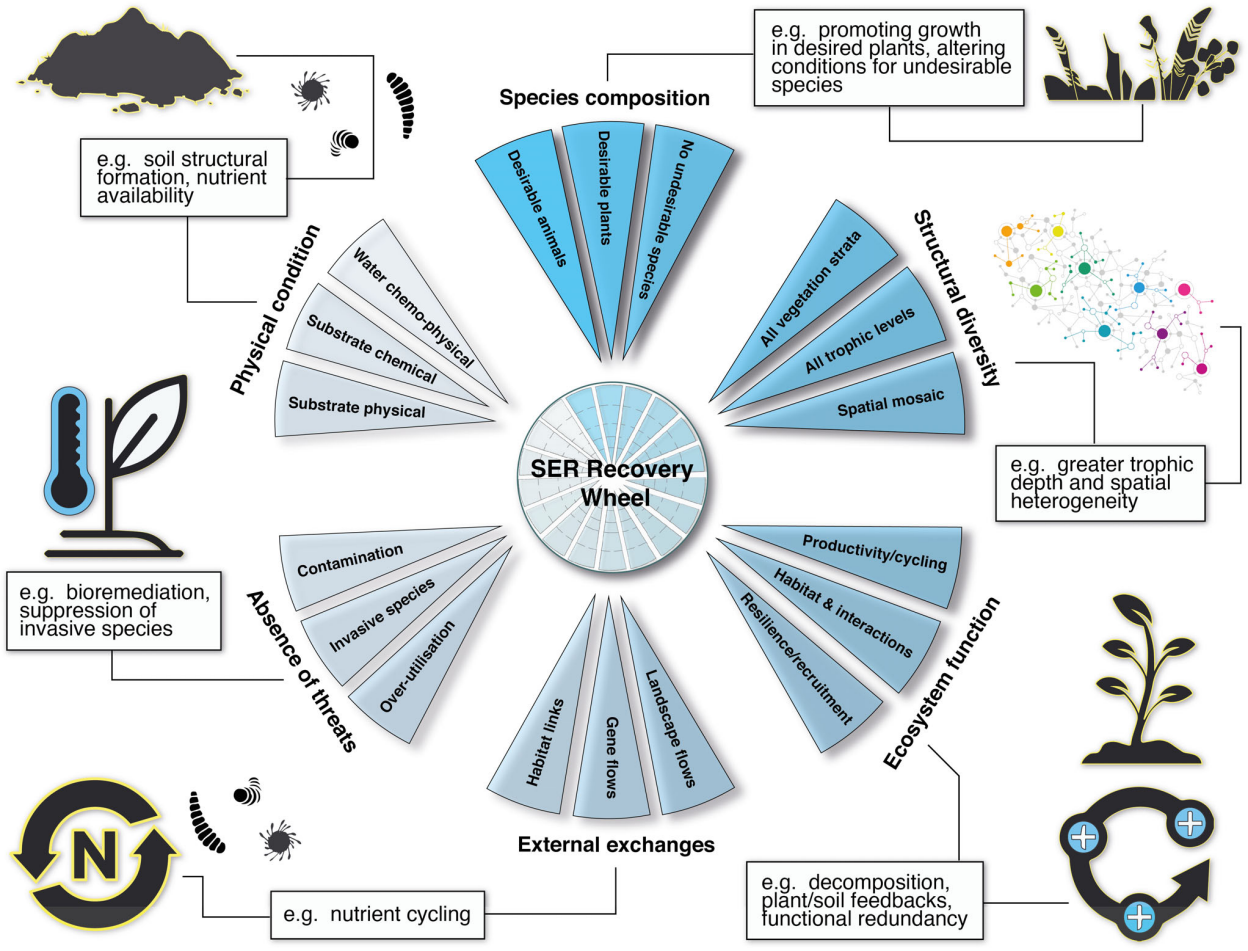
The Society for Ecological Restoration (SER) recovery wheel (Gann *et al*., 2019) and how improved integration of soil microbiota into the planning, intervention, and monitoring phases of ecosystem restoration projects could contribute to each of the six recovery outcome themes.

The essential role of soil in ecosystem restoration is recognised, mainly by considering soil physical and chemical processes in ecosystem recovery (Costantini *et al*., 2016; Muñoz‐Rojas, 2018; Perring *et al*., 2015). Over the last 15 years however, increased attention has been given to soil microbiota – the communities of bacteria, archaea, fungi, viruses and protists within soils – and their interactions in the soil system and with aboveground biota due to their essential functional roles (Harris, 2009; McKinley, 2019; Eisenhauer *et al*., 2017). Soil microbiota are among the most biodiverse and functionally important ecosystem components and are essential to many biogeochemical processes. For example, biological nitrogen fixation by diazotrophs, nitrogen‐fixing bacteria and archaea forms the foundation of Earth's terrestrial productivity (Zhu *et al*., 2022; Vitousek *et al*., 2013) and cyanobacteria (carbon and nitrogen fixers) combine with fungi, bacteria, lichens, and other organisms to form biological soil crusts (“biocrusts”) which can stabilise soil landscapes and enhance water availability (Weber *et al*., 2022; Yan‐Gui *et al*., 2013). Furthermore, soils are home to over half of Earth's biodiversity (Anthony, Bender & van der Heijden, 2023) and belowground microbial biomass is often comparable in scale to aboveground plant or animal biomass (Fierer, 2017). Soil microbiota also interact with aboveground ecosystem components and are intimately involved in plant and animal health, and *vice versa*. For example, the relationship between plants and arbuscular mycorrhizal fungi is one of the oldest terrestrial symbiotic interactions (Field & Pressel, 2018; Tisserant *et al*., 2013) where plants depend on fungi to gather essential nutrients in exchange for carbohydrates. Consequently, we can expect reciprocal shifts in above‐ and belowground ecosystem components (Kardol & Wardle, 2010; Prober *et al*., 2015). Therefore, improving the integration of soil microbiota and associated microbial ecology into ecosystem restoration will have considerable benefits across restoration planning, intervention, and monitoring phases (Fig. 1).

Historically, scientists faced technological challenges in quantifying and grasping the diversity and composition of soil microbiota, as traditional culture‐dependent methods were only able to grow <1% of microbial taxa (Alivisatos *et al*., 2015; Vartoukian, Palmer & Wade, 2010). However, modern sequencing technologies enable a detailed taxonomic and functional understanding of soil microbiota. For example, the now routine high‐throughput amplicon sequencing of DNA extracted from soil samples can provide a detailed taxonomic view of the microbiota within a given sample (Berg *et al*., 2020; Fierer, 2017). These amplicon data sets can then be associated with spatial, land‐use, environmental condition and/or restoration‐intervention data to answer ecological questions (Tedersoo *et al*., 2019; Thomsen & Willerslev, 2015; Breed *et al*., 2019).

Advances in DNA‐based technologies and improvements in our understanding of plant–soil–ecosystem interactions are enhancing our ability to use soil microbiota in restoration (Mohr *et al*., 2022). Indeed, there are several reviews on soil microbiota in a restoration context, and most have focused on theoretical aspects of including soil microbiota in restoration or relevant technological advancements (Coban, De Deyn & van der Ploeg, 2022; Contos *et al*., 2021; Rawat *et al*., 2022). Here, we complement these previous reviews by focussing on the practical interface of soil microbiota and ecosystem restoration and highlight key knowledge gaps that are limiting effective integration of soil microbiota into restoration. We highlight where and how the integration of soil microbiota has successfully occurred and identify opportunities and challenges for improved integration to enhance restoration outcomes.

## II. SOIL MICROBIOTA IN RESTORATION PLANNING

Planning a restoration project requires setting realistic goals, making informed choices of interventions, and deciding on indicators to monitor progress towards stated goals (Hobbs & Norton, 1996; Collen & Nicholson, 2014; Suding *et al*., 2015). Unfortunately, despite enormous growth in the scope and scale of restoration globally, many projects fail to achieve their stated goals despite the growing scientific rigour of restoration practice (Crouzeilles *et al*., 2016; Wortley *et al*., 2013; Sun *et al*., 2017). The reasons for these shortfalls are numerous and include insufficient consideration of soil microbiota (Heneghan *et al*., 2008; Kardol & Wardle, 2010; Farrell *et al*., 2020). Accordingly, restoration projects should routinely consider soil microbiota early as part of their *modus operandi* – together with more traditional targets and assessments for flora and fauna. These projects will then be in a better position to determine their ecological starting place, trajectory and target – all components of best‐practice restoration (Kardol & Wardle, 2010; Heneghan *et al*., 2008; Gann *et al*., 2019). Here, we outline how and when restoration projects should plan to incorporate soil microbiota from the outset to maximise benefits to ecological outcomes while avoiding wasted resources. We also highlight that improving our understanding of how specific restoration interventions affect soil microbiota is needed to plan restoration effectively.

### (1) Considering soil microbiota and restoration goal setting

Quantifying the severity of the degradation of an ecosystem is crucial in determining the level of intervention required to meet targets (Heneghan *et al*., 2008; Chazdon, 2008). For example, when a restoration site is depleted of mycorrhizal fungi required by a target plant species (e.g. mixotrophic orchid species are entirely dependent on orchid mycorrhiza for germination), there is little sense in investing resources to establish the plant without simultaneously addressing the lack of symbiotic fungi (Koziol, Crews & Bever, 2020). Furthermore, invasive plant species in a degraded landscape can modify soil microbiota to the point that the soil environment is in an alternate state of dynamic equilibrium (Suding, Gross & Houseman, 2004; Gornish *et al*., 2020). Here, removing the invasive plants and revegetating the landscape relies on the soil microbiota to move towards a state that is more supportive of the recovering native plant community which is by no means guaranteed (Harris, 2009). A revegetation‐only approach may not overcome persistent soil legacies (i.e. altered nutrient levels from fertiliser use, altered soil structure from compaction, invasive species, undesirable biological communities) and risks perpetual states of ecological invasion (Anthony *et al*., 2019; Bell, Siciliano & Lamb, 2020). As a result, specific interventions that address invasive plants and altered soil microbiota (see Section III) need to be part of the restoration planning phase. Moreover, major disturbance to soil physical and chemical conditions (e.g. from mining, erosion, compaction, excess nutrients) will alter the foundational habitat for soil microbiota, so addressing limiting abiotic factors also represents a key priority in restoration planning (Robinson *et al*., 2024). There is immense value in setting early goals to understand soil microbial ecology at the initial stages of a restoration project. This goal‐setting process will help the restoration practitioner to quantify and pre‐empt biotic and abiotic constraints or opportunities (e.g. a lack of mycorrhizal fungi, plant‐associated pathogens for structuring plant communities, altered soil physical or chemical properties).

If barriers to recovery are not identified as part of the planning stage, ecosystem recovery will likely be inhibited (Hobbs & Norton, 2004). Practitioners should address these constraints in a restoration project by, for example, using knowledge of plant–soil feedbacks in the planning phase. Restoration projects could promote negative feedbacks between plant and soil communities by, for example, inoculating sites with late‐succession soil microbiota that encourages vegetation diversity in the early recovery phase (Carbajo *et al*., 2011; Kardol, Martijn Bezemer & Van Der Putten, 2006). This can lead to mycorrhizal fungi outpacing bacterial pathogens, potentially promoting community evenness in late‐succession plants (Fierer, 2017; Kardol & Wardle, 2010). Integrating soil microbial ecology knowledge into predictive ecological frameworks (e.g. modelling different environmental change scenarios, including microbiota assembly and functional dynamics) could further allow targeted site‐specific restoration plans (Eviner & Hawkes, 2008).

Reference site selection and assessments are central elements of planning and defining goals in a restoration project (Gann *et al*., 2019). Soil physical and chemical conditions, together with plant diversity and other factors in reference sites, shape microbiota development (Fierer, 2017). While reference site soil microbiota are increasingly used in restoration monitoring (see Section IV), they are not routinely assessed during the planning phase. Gaining information on the composition, and even functional characteristics, of microbiota in both degraded and target reference sites will position projects better to tailor their interventions to address varied levels of degradation in the whole ecosystem. Soil microbiota are highly heterogeneous across even small (<1 cm^2^) spatial scales (Fierer, 2017) and therefore reference site selection and sampling design are crucial to capture variation adequately (van der Heyde, Bunce & Nevill, 2022; Liddicoat *et al*., 2022). This high level of spatial variation can impact assessments of community composition and function and distort interpretations of the reference community (Peddle *et al*., 2022).

The numbers and locations of reference site samples should account for vegetation and soil heterogeneity to provide the best possible picture of microbiota targets (Peddle *et al*., 2022; van der Heyde *et al*., 2022). One option is to implement a stratified random sampling scheme. This approach involves dividing the study area into distinct strata based on relevant factors influencing biodiversity distribution, such as vegetation and soil types or topographical features. Within each stratum, random sampling points are selected to ensure representative coverage of the area while minimising bias and distortions caused by heterogeneity. Additionally, employing systematic sampling techniques, such as grid or transect sampling or pooling samples to account for landscape heterogeneity (Bissett *et al*., 2016) can further enhance spatial representativeness and accuracy of biodiversity assessments.

A key open question in integrating soil microbiota into restoration is: what do “good” soil microbial communities look like in terms of species composition and/or functionality? The composition of soil microbiota will vary greatly in different contexts and environments with no single “ideal” microbial community (Fierer, Wood & de Mesquita, 2021). Generally speaking, the microbial community composition most suited for any given restoration site should be informed by suitable reference sites. However, understanding the specific elements of microbial communities and drivers of microbial diversity, composition and function that can be generalised across environments will improve how and where we integrate microbiota into restoration (Liddicoat *et al*., 2024). In some cases, desirable microbiota characteristics might be informed by higher‐level functional outcomes (e.g. establishment of sensitive plants, nutrient cycling, disease suppression). Various microbial taxa have seen increased research focus on their uses for restoring particular ecosystem processes or connections. For example, plant growth‐promoting rhizobacteria have potential for their ability to improve plant growth (Radhapriya, Ramachandran & Palani, 2018; Solans, Pelliza & Tadey, 2022) and enhance germination (Domínguez‐Castillo *et al*., 2021), and arbuscular mycorrhizal fungi can promote recovery of native vegetation *via* mechanisms that enhance phosphorus uptake in plants (Koziol *et al*., 2018) and improve soil physicochemical properties (Willis, Rodrigues & Harris, 2013).

While there are “good” members of microbial communities, there are also pathogens that can be harmful to microbial communities and other ecosystem components (e.g. *Phytophthora cinnamomi* is a soil‐borne plant pathogen) (Mansfield *et al*., 2024) and restoration plans need carefully to consider the risks of inadvertently spreading harmful pathogens. Importantly however, plant–pathogen interactions may be beneficial in restoration as they also play a significant role in shaping plant diversity and community dynamics. Pathogens can influence plant diversity through various mechanisms, including selection pressure on host species and facilitation of competitive interactions (Bever, Mangan & Alexander, 2015), which may impact plant and soil community stability.

### (2) When to prioritise investment in soil microbiota

Another key question that needs to be addressed to ensure restoration is as efficient and effective as practically possible is: when will the inclusion of soil microbial data improve restoration success? While the explicit consideration of soil microbiota in restoration could arguably provide benefits to all projects, it does come with additional costs (e.g. soil sampling, DNA extraction and sequencing, complex bioinformatics) and potential risks (e.g. introduction of harmful pathogens, public or policymaker scepticism from undesirable outcomes) that need to be considered to maximise positive restoration outcomes and avoid wasted resources. Soil ecosystems are complex and highly variable both within and across sites which means a one‐size‐fits‐all recommendation is problematic. Furthermore, soil microbiota are unlikely to be the only factor hindering restoration outcomes. Restoration projects should therefore include risk assessments and cost–benefit analyses on a case‐by‐case basis to determine if, and to what extent, soil microbiota should be included. The inclusion of soil microbiota in any given restoration project and any determination on the likelihood of that inclusion translating into cost‐effective improved restoration outcomes will be largely dependent on the project's goals and level of degradation or disturbance of soil physical, chemical, and biological properties.

Decisions on including soil microbiota in restoration plans and interventions should be informed largely by the impact that degrading processes have had on soils and the level of investment that is available. Even short‐term disturbances to vegetation communities with minimal disturbance to soils can cause shifts in soil microbial diversity and composition (Navarrete *et al*., 2015; Qu *et al*., 2024). However, if soil physical and chemical properties remain similar to an undisturbed state, a focus on restoring vegetation communities alone may be sufficient to see the recovery of soil microbiota. On the contrary, if degrading processes have substantially modified soil physical or chemical properties, then soil biological properties will most likely be impacted as well. For example, restoration sites that were previously used for agriculture with extensive fertiliser applications can have long‐lasting nutrient legacies that persist for decades to millennia (Turley *et al*., 2020; Parkhurst, Standish & Prober, 2022).

These persistent land‐use legacies can then act as an abiotic barrier and impede the recovery of soil microbiota and present situations where restoration should plan interventions that specifically seek to overcome these abiotic constraints (Peddle *et al*., 2024). Additionally, alterations in soil pH, moisture, and structure resulting from degradation can also influence microbial community composition and activity, further emphasising the relevance of soil physicochemical assessments in guiding the inclusion of soil microbiota in restoration initiatives. Physical and chemical conditions are generally easier to observe and test than soil microbiota and should be considered to provide as near‐optimal conditions as possible with reference sites as a guide. This sets the foundation for development of biological communities (Robinson *et al*., 2024). Restoration planning can, of course, consider “in‐principle” influences on (and *via*) microbiota, however, practitioners will be blind to actual effects and recovery if relevant attributes of microbiota remain uncharacterised. By integrating assessments of soil physical, chemical, and biological properties, restoration practitioners can tailor decisions on the inclusion of microbiota‐based interventions (see Section III) to the specific needs of degraded ecosystems, facilitating more effective restoration outcomes.

### (3) Improving conclusions on causation in soil microbiota restoration

To determine better the level of effort required to affect recovery of soil microbiota it is important to improve our understanding of how soil microbiota responds following traditional restoration interventions such as revegetation. If revegetation alone largely leads to recovery of soil microbiota, then costly assessments and interventions focussed on microbiota are probably not needed. However, attributing soil microbial recovery solely to revegetation without properly ascertaining causation will lead to soil microbiota being overlooked and risks missing opportunities either to address this crucial ecosystem component directly (Lem *et al*., 2022) or to utilise soil microbiota more as drivers of change as opposed to solely passengers (Harris, 2009). Observational studies of soil microbiota following revegetation often indicate that soil microbiota in restoration sites resemble reference sites more closely with increasing time since restoration (Barber *et al*., 2017; Gellie *et al*., 2017; Klopf *et al*., 2017; Ngugi *et al*., 2018; Parsons *et al*., 2020; Sun *et al*., 2017; Yan *et al*., 2019; Banning *et al*., 2011). These studies are often used to infer that the restoration intervention (e.g. native plant revegetation) is *causing* the restoration of soil microbiota but further rigour is needed to improve our knowledge of causal mechanisms affecting the recovery of soil microbiota.

Observational chronosequence‐based studies often suffer from unmeasured or unaccounted factors that can confound results and cloud conclusions. Other soil characteristics (both biotic and abiotic), climate, aboveground biological influences (e.g. vegetation, land‐management history), topographic relief, parent geological materials, age of development, and spatial location (e.g. proximity to external influences) will influence soil microbiota composition (Delgado‐Baquerizo *et al*., 2020; McBratney, Santos & Minasny, 2003; Pino *et al*., 2019) and may vary independently of a restoration intervention. Furthermore, restoration methods may change over time; for example, an unpredictable supply of seed resources may cause temporal variation in revegetation (Broadhurst *et al*., 2016; Ladouceur *et al*., 2018), or there may be inter‐seasonal changes in climate, or changes in revegetation practices or planting crew. These time‐dependent changes to restoration practice can introduce uncontrolled variation across the chronosequence and must be considered during chronosequence studies. In many situations, collecting sufficient covariate data to explain fully (or develop models to account for) soil microbiota spatial autocorrelations is impractical. Therefore, ensuring that these unmeasured or unaccounted influences do not compromise experimental designs and sampling plans by having appropriately designed studies is necessary.

Despite their limitations, chronosequence designs are useful for inferring ecological responses to restoration interventions through time without long‐term sampling or controlled experiments (Walker *et al*., 2010). However, explicitly planning to embed good quality experiments – such as those with adequate replication, controls and randomisation – into restoration projects will help to alleviate issues with spatial autocorrelation (van der Heyde *et al*., 2022) or pseudo‐replication (i.e. treatment *N* = groups of 1) and assist in minimising the effects of confounding factors (e.g. changes in restoration planting methods, seed supply, climate variation, spatial location). However, it should be noted that truly longitudinal and/or manipulative studies are needed to produce high‐quality evidence and conclusive support on causation (Lem *et al*., 2022) (see Section II.5).

### (4) Embedding soil microbiota experiments

**Fig. 2:**
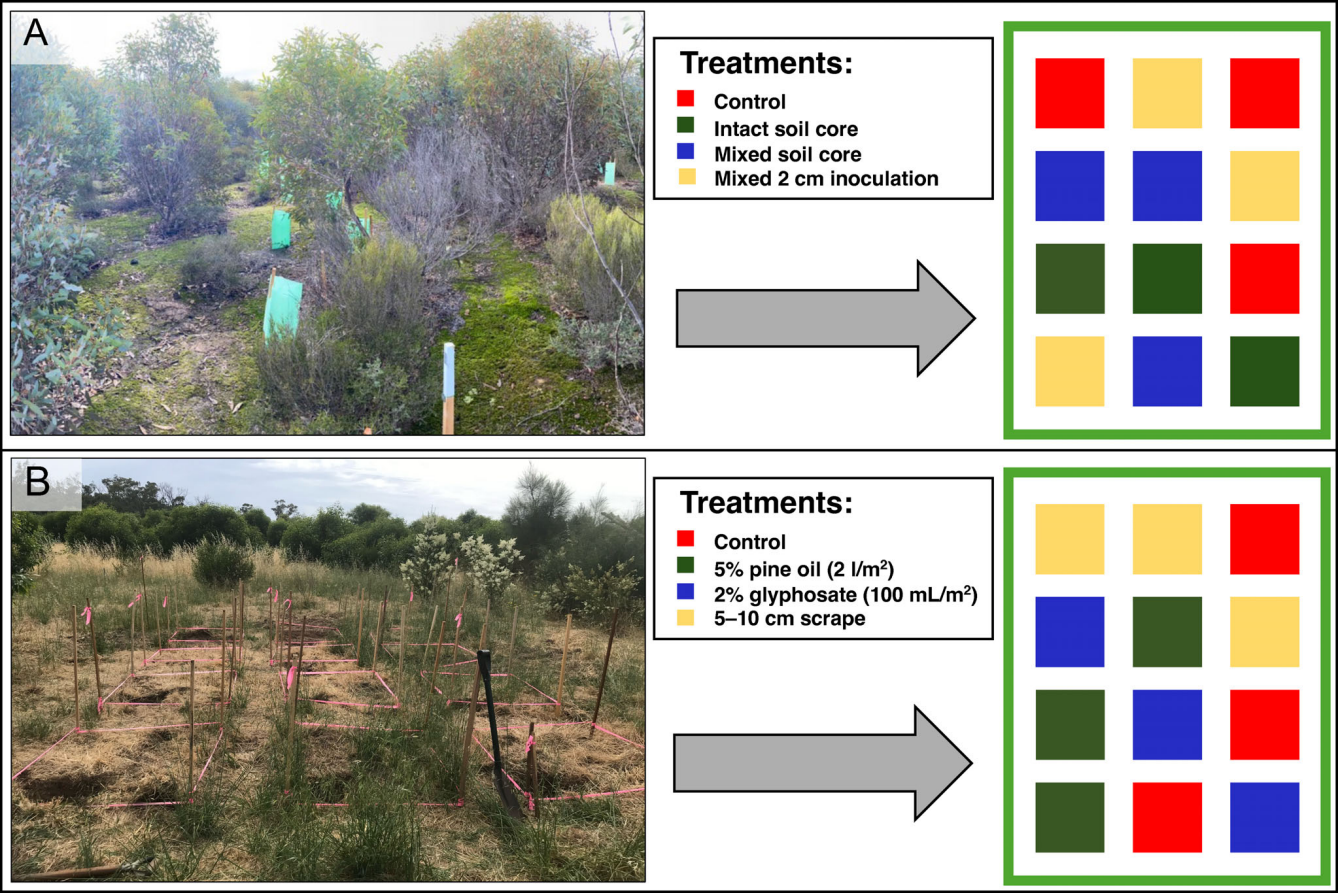
Embedding soil microbiota experiments into restoration sites. (A) A soil microbiota translocation field experiment embedded into an ongoing restoration project in Western Australia (photograph credit: Shawn Peddle). (B) Embedded soil microbiota experiments in a restoration site in the Mt Lofty Ranges, South Australia (photograph credit: Tarryn Davies). Designing and embedding experiments into restoration projects will allow for improved causal conclusions in testing microbiota‐focussed hypotheses.

By planning to embed well‐designed experiments into restoration projects, practitioners and researchers could form partnerships to address many of the limitations of chronosequence (i.e. space‐for‐time) designs (Broadhurst *et al*., 2023). Embedded experiments could nest replicated soil microbiota interventions (e.g. different soil inoculation methods or revegetation techniques) within reference and restoration sites or include spatially independent and replicated restoration interventions across a project (Fig. 2). Such an approach will improve the evidence base of the effect of specific restoration interventions on the recovery of soil microbiota and their associated functions.

Adequately replicated, randomised, controlled and comparable restoration sites are not often routinely present in restoration projects unless planned for from the outset. This lack of core scientific design principles in systems that are often used in observational studies limits conclusions that can be drawn from such research. For this reason, restoration projects could improve our evidence base by collaborating with researchers and embedding microbiota‐focussed experiments into restoration projects. Well‐designed longitudinal studies that repeatedly sample the same restoration sites through time will provide more robust evidence on cause–effect relationships than cross‐sectional chronosequence studies alone (Christie *et al*., 2019; Lem *et al*., 2022). Embedding experiments would also help close critical restoration knowledge gaps, such as knowing when a focus on soil microbiota will substantially improve restoration success. However, by their very nature, longitudinal studies require years of research and given the urgency required to address the biodiversity crisis, statistical modelling methods (e.g. structural–causal modelling, see Section II.5) will be useful to help understand the key knowledge gaps that need to be addressed with on‐site long‐term experiments and what can be solved with observational study designs alone.

### (5) Modelling approaches to ascertain causation

Where particular microbiota‐oriented outcomes are desired, but restoration activity cannot wait for definitive experimentally derived knowledge on cause–effect relationships, certain modelling approaches may help to distil information from relevant existing microbiota‐restoration data sets. Techniques such as structural–causal modelling, structural equation modelling and path analysis can be applied to test hypotheses using observational cross‐sectional data (Arif & MacNeil, 2023; Grace & Irvine, 2020). These approaches involve specifying a theoretical model that reflects likely causal relationships among variables of interest, including both observed and latent (i.e. unmeasured) variables. Then, the hypotheses are tested by specifying directional paths that represent the assumed causal relationship between variables in the model. Using the observed data, parameters (i.e. coefficients) of the specified model are estimated. These estimates assess the strength of the hypothesised causal/directional relationships and goodness of fit metrics indicate how well the model aligns with the data, even in the absence of experimental evidence (Eisenhauer *et al*., 2015), facilitating informed decision making in restoration planning.

As a specific example, we could consider a scenario where a restoration intervention aims to enhance soil fertility and plant growth by introducing specific microbial species or communities. By using structural causal modelling, researchers can construct a theoretical model that includes variables related to soil microbiota composition and function, soil fertility, and plant performance. They can hypothesise directional paths between these variables representing the assumed causal relationships. Through analysis of observational cross‐sectional data from similar restoration projects and controlling for covariates, researchers can estimate the parameters of the model and assess the strength and direction of the hypothesised causal relationships. For instance, it might be that certain microbial taxa or functional genes are strongly associated with increased soil fertility, which in turn positively impacts plant growth.

While structural–causal modelling with cross‐sectional data is powerful, it has limitations. It cannot establish causality as definitively as controlled or longitudinal experiments, and causality may be more challenging to infer in the presence of unobserved confounders especially in systems such as soil with thousands of distinct taxa and many functional groups (Eisenhauer *et al*., 2022). However, it allows researchers to specify, estimate and evaluate complex causal models providing insights into causal relationships (e.g. the relative roles of physical, chemical and biological properties of soil in limiting soil ecosystem recovery; the facilitation or following roles of soil biota and aboveground plant and animal communities during restoration; the influence of climate change and other major global degrading forces on limiting the recovery of soil ecosystems) without the need for experimental or longitudinal designs. Furthermore, the strength of causal claims should always be considered in the context of the study's design and the potential presence of unobserved confounding variables.

## III. RESTORATION INTERVENTIONS THAT DIRECTLY TARGET SOIL MICROBIOTA

It is possible to manipulate soil microbiota to assist in the recovery of degraded ecosystems by reinforcing beneficial interactions between plant species and soil microbiota lost through degradation (Aghili *et al*., 2014; van der Putten *et al*., 2016; Albornoz *et al*., 2022). Soil microbiota‐focussed interventions can improve plant species prospects by improving plant growth, and depending on the ecological system, diverse soil microbiota have also been shown to mediate vegetation community diversity and improve ecosystem productivity (Naeem *et al*., 1994; Yang *et al*., 2021). Informed by mechanisms of ecosystem recovery and species‐specific responses, soil microbiota interventions can advance restoration objectives and restore the diminished capacity of impacted ecosystems to recover naturally. In this section, we review more established (e.g. soil inoculations) and less well‐established (e.g. specific microbial cultures, seed enhancements) ways to manipulate soil microbiota to improve restoration outcomes. We note that obvious abiotic barriers to the development of site‐specific favourable soil microbial communities (e.g. soil substrate problems, excess nutrients, low pH, high salinity) should be identified and addressed before attempting direct manipulation of soil microbiota.

### (1) Whole‐soil translocations and microbial inoculations

Translocating whole soil communities – whether in the form of intact turfs or homogenised bulk soil – is one way of inoculating soil microbiota into degraded ecosystems to shift the microbial community towards one that is more representative of a target ecosystem. This essentially involves collecting soil from a reference ecosystem and translocating it directly into a restoration site (Koziol *et al*., 2018; Wubs *et al*., 2016; Carbajo *et al*., 2011). Inoculating degraded sites with reference ecosystem soil and associated biota has been shown to improve the growth and establishment of desirable native plants and exclude weeds in both greenhouse and field conditions (Koziol *et al*., 2018; Wubs *et al*., 2016; Fahey & Flory, 2022). For example, Wubs *et al*. (2019*a*) showed that soil inoculations can have ecosystem legacy effects that steer successional changes and can last for at least two decades. Importantly, however, Gerrits *et al*. (2023) highlight how the directionality of this legacy effect depends on the suitability or fit of translocated soil to the recipient site, with mismatches steering communities in the wrong direction. Similar interventions can also shift the direction of the development of vegetation communities (Wubs *et al*., 2016) and improve prospects for native vegetation success (Wubs *et al*., 2019*b*). However, while research has shown a benefit for the restoration of vegetation, few studies have focussed on the efficacy of soil translocations to shifting whole microbial communities themselves.

Substantial knowledge gaps remain on the effectiveness of soil translocations, including: what methods are most effective (e.g. bulk soil, intact turfs, volumes required), to what extent do soil physical and chemical properties in recipient sites impact establishment, how do priority effects impact on microbial community recovery (i.e. establishment may be dependent on the order of arrival of specific taxa), and, how does the coalescence of distinctly different soil communities impact successful establishment? As such, further research on whole‐soil translocations and inoculations should focus on addressing these knowledge gaps *via* embedded experiments to understand better how soil volume, translocation method, and community coalescence dynamics affect microbial community assembly across varied ecosystems and soil types. Addressing these knowledge gaps will then enable the research community to develop decision‐support frameworks to help determine when whole‐soil translocations will provide restoration benefits that are commensurate with cost.

Another critical open question relating to soil translocation is: how can we minimise the impacts soil translocations have on donor ecosystems? While soil translocations may be effective, soil collection can impact remnant habitats and consideration is needed to limit impacts to remnant sites while providing a benefit to degraded sites. Solutions are needed to scale up soil translocations outside situations where soil can be harvested because existing remnant habitat is already being cleared. As such, decisions on interventions impacting remnant habitat will need to weigh factors such as the contribution of remnant habitat to support the integrity and viability of restoration or conservation efforts (Tulloch *et al*., 2016; Wintle *et al*., 2019), or if a degree of destructive harvesting of soil resources from remnant sites can provide restoration benefits that outweigh impacts to remnant habitat. To address the need for reliable seed sourcing in restoration or revegetation, seed‐production areas are being established instead of relying on sourcing seeds from remnant habitats (i.e. target plants are grown *ex‐situ* “en masse” to produce seed stock) (Zinnen *et al*., 2021). This concept could potentially be applied to soil microbiota with soil microbiota production areas, although various open questions (i.e. how do we cultivate whole target microbial communities, can we subset communities to focus on particular taxa, and what is the “ideal” composition of these communities) need to be addressed before soil microbiota production areas can be effectively implemented at scale.

Despite these knowledge gaps, whole‐soil translocations are increasingly used in large‐scale restoration projects where topsoil is salvaged as part of the initial disturbance (e.g. surface strip mining) and then reinstated during restoration (Tibbett, 2010; Schmid *et al*., 2020; Liddicoat *et al*., 2022). The objective of topsoil transfer is to preserve the soil‐stored seedbank rather than the soil microbiota *per se*. Still, benefits from the reservoir of microbiota contained in these topsoils present an opportunity to improve restoration outcomes. Limiting the amount of time for which soils are stockpiled before translocation is crucial as stockpiling can disrupt biological integrity and impact microbial diversity and composition (Hernandez *et al*., 2024; Valliere *et al*., 2022). In best‐practice cases, the direct return of harvested topsoil to nearby restoration sites will limit the physical and biological degradation of soil from long‐term stockpiling (Rokich *et al*., 2000; Peddle *et al*., 2022). However, the impact of the collection and homogenisation of vertical soil profiles during the transfer process on soil microbiota is likely detrimental but still poorly understood.

**Fig. 3:**
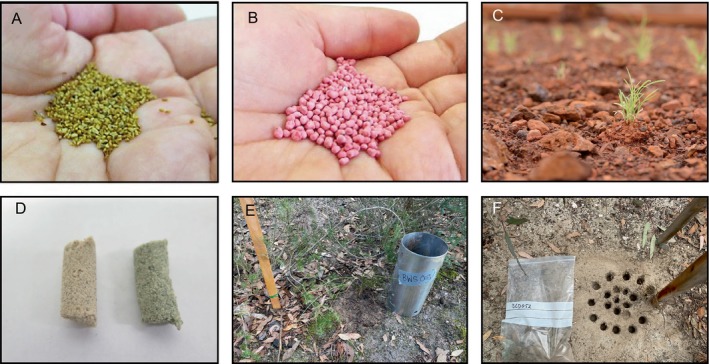
Manipulating soil microbiota as part of restoration interventions. *Eucalyptus* seeds (A) before and (B) after coating with mycorrhizae inoculants (photograph credit: Todd Erickson). (C) Seedlings germinated from extruded seed pellets containing live soil (photograph credit: Todd Erickson). (D) Control (sand + bentonite, left) and cyanobacteria encapsulated pellets (right) (photograph credit: Miriam Muñoz‐Rojas). (E, F) Whole soil translocation experiment with (E) translocated intact soil core and (F), resampling 1 year after translocation to assess establishment and dispersal of soil microbiota (photograph credit: Shawn Peddle).

An emerging approach that avoids broadacre spreading of whole soil is the targeted use of microbiota from local soils *via* extruded pellets or coatings as a vessel for seed delivery (Gornish, Arnold & Fehmi, 2019; Madsen *et al*., 2016) (Fig. 3). This method is designed to improve the precision of seed delivery in large‐scale restoration efforts that simultaneously provide beneficial soil microbiota and the target seeds. Such an approach can reduce the demand for soil by 100‐fold (Stock *et al*., 2020). However, similar questions to those identified earlier in relation to whole‐soil inoculations are still unresolved and applicable here. For example: how do the mechanical and chemical disturbances of creating the pellets affect microbial community composition, and how well can targeted microbial communities in extruded pellets be established within donor soils with vastly different microbial communities or physicochemical properties?

### (2) Seed enhancements that contain microbial additives

Seed enhancements that add specific microbial inoculants in a restoration context can improve the germination and growth of desirable plant species (Chua *et al*., 2019; Dadzie *et al*., 2023; O'Callaghan, 2016). Seed coating involves the precise application of binders and mineral powders to seeds to create a thin artificial layer of material capable of altering the physical shape of seeds and/or carrying beneficial products such as microbiota (Fig. 3A–C) (Brown *et al*., 2021; Erickson *et al*., 2021). Similarly, extruded pellets made *via* extrusion or moulding technologies can make larger seed‐soil matrices while offering the same microbial inoculation opportunities. For instance, microbiota can be added to the seed coat and/or into extruded pellet ingredients either dry within the powder or wet *via* the binder (Alfonzetti *et al*., 2023; Dadzie *et al*., 2023; Munro *et al*., 2024). Alternatively, seed priming involves immersing seeds in water‐based (i.e. hydro‐priming) or osmotically controlled (i.e. osmo‐priming) solutions or soil matrix (i.e. matrix‐priming) to commence the process of germination under controlled conditions, followed by a re‐drying step (Brown *et al*., 2021; Madsen *et al*., 2018). Once sown, primed seeds show a much higher and more rapid germination potential, recruitment synchronicity and seedling vigour. Priming with additives like microbial suspensions can facilitate the uptake of beneficial microbiota directly onto and potentially into the seeds (O'Callaghan, 2016; Muñoz‐Rojas, 2018). Consequently, the targeted microbiota will be established directly in the soil near the germinating seed or within the seedling tissue itself (O'Callaghan, 2016; Chua *et al*., 2019).

There have been successful implementations of naturally obtained microbiota that benefit key restoration plant species by seed enhancements (including extruded seed pelleting and/or hydro‐priming with microbial additives) (Muñoz‐Rojas *et al*., 2018; Dadzie *et al*., 2022). The improved accessibility and effectiveness of these techniques represent a valuable opportunity for restoration. Bioencapsulation – which involves encasing seeds in protective polymers (natural and/or synthetic) that sustain microbial inoculants – can also be used to enhance the survival of microbiota during storage and introduction into field soils (Brown *et al*., 2021; Schoebitz, López & Roldán, 2013). These methods may be more suitable for large‐scale field settings through the gradual and prolonged release of target microbiota. These extruded pellet or seed coatings can be applied widely *via* targeted dispersal across restoration sites to improve seed vitality and establish beneficial and targeted microbes, such as cyanobacteria for forming soil biocrusts (Román *et al*., 2020). Additionally, using materials such as clay, carbohydrates and/or hydrogels to encase seeds and engineer seed microenvironments represents a recent technological transfer into restoration from the agricultural and pharmaceutical sectors, and shows promise for improving restoration success *via* the incorporation of microbiota (Zvinavashe *et al*., 2019). Still, limitations preventing the implementation of these approaches include the challenge of identifying appropriate inoculants across different ecological communities, accumulating sufficient biomass, and testing these applications under field conditions. Field testing must be improved before this technology can provide a reliable and scalable tool for restoration practitioners (Ayuso *et al*., 2017; Román *et al*., 2018).

### (3) Microbial cultures and suspensions

Microbial cultures and suspensions are additional alternative and promising lab‐based approaches to whole‐soil inoculations and seed enhancement technologies. They rely on less‐destructive sourcing of soil microbiota requiring smaller quantities of soil and, therefore, have a reduced impact on reference ecosystems compared with whole‐soil translocations (Dadzie *et al*., 2022; Muñoz‐Rojas *et al*., 2018; Román *et al*., 2020). Microbial cultures can be isolated into a liquid medium *via* suspensions. These suspensions and cultures offer versatility: they could introduce specific microbiota into liquids or pastes and inoculate plant tissues like roots, seeds, or soils directly (Olle & Williams, 2013; Vassilev *et al*., 2020). Isolation and culturing of soil microbiota within the context of native plants has resulted in increased recruitment and growth of a variety of plant taxa [e.g. mangroves, legumes, cacti (Bacilio, Hernandez & Bashan, 2006; Bashan *et al*., 2012; Radhapriya *et al*., 2018)]. Expected applications of strain‐culturing could further improve the cultivation and recovery of rare and endangered plants (Dutta, Na & Lee, 2021) and are commonplace within orchid fungal symbiosis research (Hossain, 2022). However, the successful establishment of inoculants is highly dependent on priority effects, niche/resource availability, and community cohesiveness (Debray *et al*., 2021; Diaz‐Colunga *et al*., 2022). Limitations to these approaches include their poor shelf‐life and the inadvertent loss of desirable microbiota (Ramakrishna, Yadav & Li, 2019). Moreover, many mass‐produced inocula are likely unsuitable for natural environments (Koziol *et al*., 2018; Jiang *et al*., 2023; Kaminsky *et al*., 2019). These techniques, therefore, require additional investment to improve their reliability, affordability, and broad‐scale application if they are going to be of general use in restoration. In addition, the cost and technical challenges of matching isolated cultures and suspensions to a specific restoration context is a hurdle to overcome.

### (4) Targeting specific microbiota

Specific microbial taxa can be lacking in an ecosystem, disproportionally impacting plant fitness (Thrall *et al*., 2001). For example, obligate symbionts (e.g. rhizobia) often fail to persist in degraded soils since their survival relies on the presence and persistence of their host plant species (Thrall *et al*., 2001; Berruti *et al*., 2016) or other microbes within a whole community. For bulk soil inoculations, microbiota specificity is low as this approach relies on a whole‐of‐community transfer. Therefore, varying degrees of specificity are relied upon for microbial cultures and suspensions when targeted for use as an additive in direct soil applications or *via* priming, coating and extruded pelleting approaches. Because of this variable specificity, the required level of microbe–host matching is an important factor to consider when developing soil microbiota interventions (e.g. does an inoculum need to land precisely within the root zone of a target plant to succeed?). Furthermore, reliance on expensive and highly technical approaches could be a liability for restoration practice where patents and corporate control of technology could limit affordable uptake and equitable use of tools needed to improve restoration outcomes (Osborne *et al*., 2021).

A targeted consortium of microbes (e.g. multiple taxa of cyanobacteria) may be preferable over individual strains (Chua *et al*., 2019; Dadzie *et al*., 2022), especially since a diverse community should result in more resilient microbiota (Chua *et al*., 2019; Rodriguez & Durán, 2020; Berendsen *et al*., 2018). Culturing diverse microbial consortia can be challenging however, as varying capture and growth rates across taxa are likely (Kaminsky *et al*., 2019). A further roadblock is selecting the appropriate techniques to capture, extract and transfer the targeted microbiota or strains. This will be particularly challenging for obligate symbionts, which can be particularly hard to isolate and culture (Berruti *et al*., 2016).

The use of plant hosts has been proposed as a way to culture a targeted microbiota. Trap cultures in soil, for example, involve collecting soil samples containing target microbial communities – such as arbuscular mycorrhizal fungi – from whole soil in a reference ecosystem, which is then propagated with host plants *ex‐situ* for later inoculation (Koziol *et al*., 2018). Techniques like this could be scaled up in soil microbiota production areas which could reduce impacts on remnant ecosystems comparatively to the direct transfer of topsoil. However, these soil‐culturing systems require substantial time and technical investments to establish them. Once operational, communities may shift away from their “wild type” or desired community. Evidence suggests that these communities can change to undesirable states over time, due to the build‐up of soil pathogens (Bauer, Mack & Bever, 2015) or reduced diversity within the microbial communities, which could harm host plants (Trejo‐Aguilar *et al*., 2013) undermining the effectiveness of microbial products. Alternatively, harnessing the positive soil legacies of plants and host‐mediated microbiome engineering have been proposed as methods of selecting for specific functional outcomes in microbial communities by subjecting plants to specific selective pressures (e.g. inducing drought tolerance in a host‐plant's microbiota *via* instigating water stress) (Mueller & Sachs, 2015; Pineda, Kaplan & Bezemer, 2017; Gopal & Gupta, 2016). However, our ability to introduce targeted microbiota or a select microbial strain depends on our capacity to identify specific taxa of interest and extract, propagate, and successfully re‐introduce them effectively and in a replicable way.

### (5) Promoting positive soil legacies

Utilising the positive soil legacies of plants could improve the fitness of plant species used in revegetation (Chua *et al*., 2019; Koziol *et al*., 2018). Positive soil legacies involve the recruitment and fostering of favourable microbiota in the soil through a preparatory generation of plant growth, thereby priming the soil microbiota to provide a plant fitness advantage for the next generation (Pineda *et al*., 2017; Gopal & Gupta, 2016).

The potential for creating positive soil legacies through priming the soil with specific plants has been demonstrated with the wildflower *Senecio jacobaea* (Pineda *et al*., 2017). When exposed to insect pests, this plant generated a feedback mechanism where sugars and organic acids exuded from its roots maintained a distinct soil fungal community that affected the regulation of amino acids in the host plant's phloem sap, providing the plant with reduced herbivore populations (Kos *et al*., 2015). Also, Buchenau, van Kleunen & Wilschut (2022) observed that some European grasses could see improved growth in the second generation of plants grown in drought‐exposed and nutrient‐limited soils due to a positive legacy of the soil microbiota. The next step for utilising positive soil legacies better is to improve understanding of the generality of this effect as it is not present for all plant species (Kaisermann *et al*., 2017).

Understanding microbial‐mediated stress responses in plants and how plant–microbial interactions can be applied to improve plant stress tolerance presents promising restoration opportunities (Larson, Venette & Larson, 2022; Petipas, Geber & Lau, 2021; Valliere *et al*., 2020). The transfer of soil microbiota from non‐local soils or across environmental gradients (e.g. temperature, aridity, nutrient) into revegetation sites could instil stress‐ameliorating interactions between plants and the relocated microbiota. This could build resilience to developing stress and disturbance expected under climate change or site‐specific legacies of previous land use – provided we improve our understanding of patterns of host‐plant‐specific *versus* general adapted microbial functions (Petipas *et al*., 2021).

## IV. MONITORING SOIL MICROBIOTA FOR RESTORATION

Accurate and efficient monitoring of biotic and abiotic responses to restoration is required to ensure that recovery is progressing as desired (Ruiz‐Jaen & Mitchell Aide, 2005; Collen & Nicholson, 2014; Muñoz‐Rojas, 2018) and facilitates both the evaluation of a project against its stated goals and amendments to a project should evaluation uncover a lack of suitable progress (Gann *et al*., 2019; Liddicoat *et al*., 2022). While the historical inclusion of soil microbiota in restoration goals has been rare, they are now increasingly used to assess ecosystem recovery as advances in sequencing technology improve our understanding of microbial ecology (Liddicoat *et al*., 2022; Mohr *et al*., 2022; van der Heyde *et al*., 2022). Most studies have used a chronosequence design (see Section II.3 for common issues with this design) and have characterised soil microbiota using a variety of methods (Table 1). Different microbiota assessment methods have their pros and cons, which we briefly outline below with key study design considerations.

**Table 1:** Key tools used to characterise microbial communities, functions and/or biomass in soil.

Technique	DNA based	Complexity	Data analysis	Biomass data	Taxonomic data	Functional data
Phospholipid fatty acid analysis (PLFA)	No	Moderate	Easy	Yes	Poor	Poor
Amplicon sequencing	Yes	Low	Moderate	No	Detailed	Poor to moderate
Shotgun metagenomics	Yes	High	Hard	No	Detailed	Detailed (potential)
Metatranscriptomics	Yes	Very high	Hard	No	Moderate	Detailed

### (1) Monitoring microbial diversity, composition, and function

**Fig. 4:**
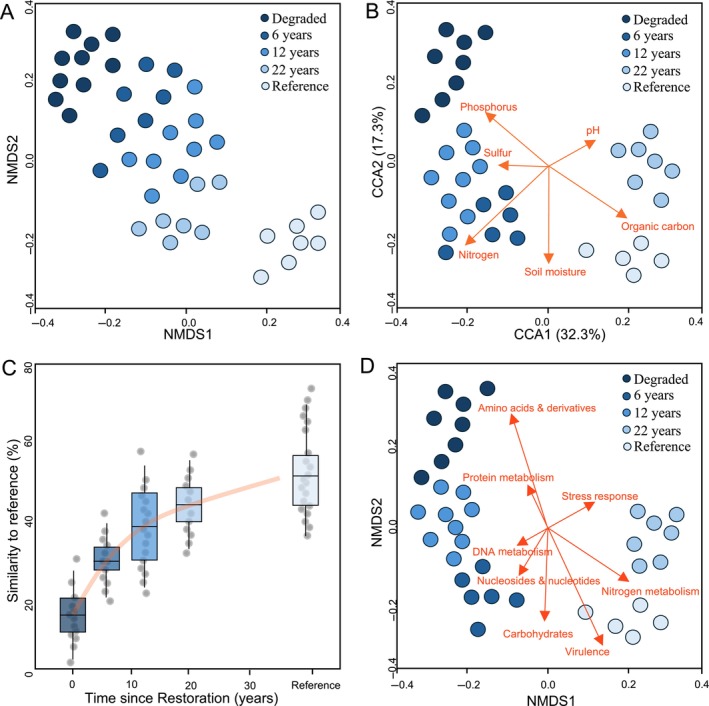
Illustrations of examples of data presentations useful for monitoring soil microbiota in restoration. (A) A non‐metric multidimensional scaling (NMDS) ordination from amplicon sequencing data comparing differences in microbial community composition across a restoration chronosequence. The ordination indicates communities become increasingly similar to reference sites with increasing time since restoration. (B) A constrained correspondence analysis (CCA) ordination indicating how microbial community composition is constrained by soil physical and chemical properties. (C) Boxplots indicating the similarity to reference sites of microbial communities across a restoration chronosequence. The box plots indicate increased similarity to reference sites with increased time since restoration and the red line indicates the projected number of years until full recovery. (D) An NMDS ordination from shotgun metagenomic sequencing data showing shifts in microbial functional gene composition across a restoration chronosequence overlaid with correlated Level 1 functional categories.

High‐throughput amplicon sequencing is an increasingly common method used to characterise microbial diversity and community composition in a sample. Amplicon‐based data can be used to assess differences in microbial communities across restoration treatments, controls, or ages (Fig. 4A, B). Since amplicon sequencing is increasingly accessible and affordable, there has been rapid, recent growth in restoration studies using this approach (Mohr *et al*., 2022). This method presents a detailed picture of microbial diversity and community composition, which is not provided by culture‐dependent methods or phospholipid fatty acid analysis (PLFA) approaches. Since phospholipids are only collected from live microbes during sampling, PLFA provides a snapshot of live microbial biomass. As such, PLFA has an advantage over DNA sequence‐based approaches where DNA is sampled from both live and dead microbes and living biomass cannot be estimated (Seymour, 2019). However, unlike sequence‐based approaches, PLFA cannot provide detailed taxonomic insights into microbial diversity or composition. Therefore, it has been recommended that combining PLFA and sequence‐based approaches can provide an accurate assessment of both live microbial biomass and community composition (Nkongolo & Narendrula‐Kotha, 2020).

Linking microbial amplicon sequencing data to microbial functions would be very useful for monitoring functional changes in soil microbiota during restoration. However, limited understanding of the functional roles of most microbial taxa, particularly in soils, presents a critical problem to the uptake of these tools (Morris, Meyer & Bohannan, 2020) and should be a key priority moving forward. Moreover, horizontal gene transfer between taxa would further confound amplicon‐based taxonomy and functionality linkages, and as such, inferring function from amplicon data is not currently advised (Morris *et al*., 2020; Makiola *et al*., 2020). Some bioinformatic tools can estimate functionality from amplicon sequence data, such as PICRUSt2 (Douglas *et al*., 2020) and Tax4Fun2 (Wemheuer *et al*., 2020). However, there are many limitations with these approaches including that predicted functions represent approximations (not exact matches to the reference functions), and functional predictions are biased towards existing reference genomes and less likely to provide good functional characterisations for understudied environments such as soil (Douglas *et al*., 2020; Sun, Jones & Fodor, 2020).

Growing opportunities from shotgun metagenomic and/or metatranscriptomic data sets are now available to provide high‐quality insights into microbial functions in a restoration context (Breed *et al*., 2019; Sun *et al*., 2020). Shotgun metagenomics is similar to amplicon sequencing, but instead of amplifying a targeted gene region, it involves random sequencing of all DNA from within a sample. These random fragments of the metagenome within a sample can be aligned to functional and taxonomic databases and/or assembled into metagenome‐assembled genomes (MAGs). These approaches can provide functional gene abundance data directly instead of just taxonomic data or inferred functions from amplicon data. Restoration scientists can then interrogate these functional gene abundance data for functions of interest – such as genes associated with nitrogen fixation or primary productivity – and compare these before and after restoration to assess changes to key ecological functions and processes (Sun & Badgley, 2019). Importantly though, metagenomics involves a much higher sequencing cost, and the complexity of data processing and analysis requires expertise that could place a disproportionate burden on restoration projects. Furthermore, both amplicon and shotgun metagenomics do not discern between active and inactive organisms as relic DNA in the sample is also sequenced (Li *et al*., 2017; Sun & Ge, 2023).

An additional layer of functional information can be obtained by collecting, isolating, and sequencing RNA (as opposed to DNA used in amplicon and metagenomic approaches) from a soil sample with metatranscriptomics. This technique can be a powerful asset in studying soil ecosystem services carried out by microbiota but is not yet widely used in ecological contexts (Breed *et al*., 2019). Analysing total community RNA transcripts can potentially reveal a microbiome's gene expression under specific conditions, known as the active functional profile. This approach provides an opportunity to study direct alterations of the (meta‐)transcriptome in response to different environmental conditions. High functional redundancy is common in soil microbiomes (Louca *et al*., 2018; Prosser, 2020) and identifying relationships between microbial community structure and function remains challenging because observed community functions are often difficult to link to specific taxonomic groups. Furthermore, RNA‐based methods are generally more expensive and time‐consuming than DNA‐based methods (Cordier *et al*., 2019), and the unstable nature of RNA molecules presents a technical challenge. Because transcriptional profiles can vary considerably over time, any information gained *via* metatranscriptomics should be interpreted as a “snapshot” in time. Nonetheless, metatranscriptomics can be a powerful asset in trying to shed light on the dynamics of ecosystem functions carried out by microbiota and warrants consideration as part of a multi‐omics approach (Aguiar‐Pulido *et al*., 2016).

### (2) Monitoring restoration trajectories with soil microbiota

Due to their essential roles in ecological processes and rapid responses to environmental changes, soil microbiota are increasingly recognised for their use as bioindicators of recovery following ecosystem restoration (Coban *et al*., 2022; Rawat *et al*., 2022). Changes in land use, vegetation composition, and soil physical and chemical properties can lead to reciprocal changes in soil microbiota (Delgado‐Baquerizo *et al*., 2020). Accordingly, microbiota depend on feedbacks with aboveground ecosystem components and monitoring changes to microbiota following restoration interventions can present a holistic indication of any potential recovery trajectory, or lack thereof (van der Heyde *et al*., 2022).

Using chronosequence‐based amplicon sequence data, Liddicoat *et al*. (2022) modelled trajectories of soil microbiota recovery towards reference states. The authors assessed whole‐soil bacterial communities and included multiple reference samples, and measures of similarity among these, to determine the level of natural variation that should be recognised when setting realistic targets for rehabilitation. Analysis of the entire community avoids reliance on any specific indicator taxa – whose presence and abundance may vary considerably with site history and current conditions. Asymptotic logarithmic models (i.e. the response variable approaches a given value with a diminishing rate of change as the independent variable increases) were used to visualise trends and predict recovery timeframes as similarity‐to‐reference values increased with rehabilitation age, approaching a nominal target defined as the median of among‐reference similarities (Fig. 4C).

Moving beyond community composition‐based metrics alone, Sun & Badgley (2019) used shotgun metagenomics to assess changes in both community composition and functional gene abundance across a mine site restoration chronosequence (Fig. 4D). The metagenomic data indicated taxonomic shifts with restored samples becoming more like the reference with increasing age as well as successional patterns in important functional groups associated with nitrate and ammonia oxidisers. The authors did however highlight that while relative abundances of methanotrophs and methane monooxygenase genes increased through the chronosequence, their levels were still much lower than those from unmined reference sites even 31 years post revegetation.

As ongoing knowledge gaps are addressed and further refinements are made to how we effectively incorporate microbiota in restoration, ensuring benefits from the use of these tools become equitable is essential to ensure society and nature benefit equally across the globe. The use of many of the tools described here and collection and dissemination of data is already skewed away from the global south. For example, the US National Center for Biotechnology Information (NCBI) nucleotide database contains no sequence‐read archives from restoration studies in South America, Africa, or much of Asia (aside from China and Japan) (Robinson *et al*., 2023). Securing adequate funding for restoration is already a barrier to restoration success and further reliance on costly technologies to effect positive outcomes will likely further increase the restoration gap between developed and developing nations. Ensuring equitable benefit from improved integration of soil microbiota is likely a more difficult task than overcoming technical barriers or knowledge gaps but is essential to effecting improvements where they are most needed.

## V. CONCLUSIONS

(1) Better integration of soil microbiota into restoration planning, interventions and monitoring has clear potential to improve ecosystem restoration outcomes.
(2) The research community should tackle several key knowledge gaps to help integrate soil microbiota into ecosystem restoration, including: (*i*) how do we best determine causation in microbial responses to restoration, (*ii*) when is the inclusion of soil microbiota worthwhile, (*iii*) what do “*good*” soil microbial communities look like and what elements can be generalised across broad restoration settings, (*iv*) what methods of inoculating restoration sites with whole microbial communities are most effective and how do we limit disturbance to remnant habitat while sourcing inoculants, (*v*) what barriers impede the establishment of targeted microbiota such as plant growth‐promoting rhizobacteria and how do we overcome them, and (*vi*) how do we ensure equitable access to these tools to benefit society and nature maximally across the globe?
(3) Further collaboration between practitioners and cross‐disciplinary researchers can help to address knowledge gaps, increase our understanding of restoration‐focused microbial processes and apply established microbiota‐focused restoration techniques and knowledge to practical on‐the‐ground applications.
(4) Experiments with spatial and temporal components embedded into restoration projects that account for confounding factors will help overcome the limitations of observational studies in an effective way to fill knowledge gaps. Modelling techniques – including structural–causal models – will be useful for developing causal understanding and informing research priorities.
(5) Whole‐soil translocations and microbial inoculations can drive successional changes in both soil microbiota and aboveground vegetation towards a target reference ecosystem; further research however is needed to understand barriers in microbial establishment and how to overcome them. To limit further degradation associated with sourcing soil inocula, these inoculations can be scaled up using various seed‐enhancement technologies that add specific microbial inoculants to seeds as a coating or extruded pellets.
(6) Positive soil legacies can be promoted in restoration sites to prime soil microbiota to provide a fitness advantage to future plant generations and instil greater resiliency to environmental stressors including climate change‐associated increases in aridity.
(7) Advancements in various ’omics technologies allow for effective monitoring of recovery trajectories to assess progress following restoration and continually adapt management strategies based on progress. Recovery of both microbial community composition and their ecologically important functions should be investigated to determine the state of the ecosystem and any further interventions needed to affect the restoration of biodiverse and functional ecosystems.

## AUTHOR CONTRIBUTIONS

S.D.P., R.J.H., R.J.B., C.D.W., S.L.K. and M.F.B. conceived the study. S.D.P., R.J.H., R.J.B., S.L.K. and M.F.B. wrote the first draft of the paper with input from all authors.
